# Penicillin G Sodium as a Treatment of Otosyphilis with Hearing Loss

**DOI:** 10.3390/antibiotics8020047

**Published:** 2019-04-28

**Authors:** Verajit Chotmongkol, Sittichai Khamsai, Patravoot Vatanasapt, Kittisak Sawanyawisuth

**Affiliations:** 1 Department of Medicine, Faculty of Medicine, Khon Kaen University, Khon Kaen 40002, Thailand; vercho@kku.ac.th (V.C.); sittichai_k@kkumail.com (S.K.); 2 North-Eastern Stroke Research Group, Research Center in Back, Neck, Other Joint Pain and Human Performance (BNOJPH), Research and Training Center for Enhancing Quality of Life of Working Age People, and Research and Diagnostic Center for Emerging Infectious Diseases (RCEID), Khon Kaen University, Khon Kaen 40002, Thailand; 3 Department of Otorhinolaryngology, Faculty of Medicine, Khon Kaen University, Khon Kaen 40002, Thailand; patvat@kku.ac.th

**Keywords:** otosyphilis, *Treponema pallidum*, treatment, penicillin, outcomes, audiogram

## Abstract

Otosyphilis is one contributing cause of hearing loss in adult patients. There are limited studies on the treatment regimens of otosyphilis. Penicillin G sodium (PGS) plus additional medications, such as benzathine penicillin and probenecid, is an effective regimen. This study investigated the efficacy of PGS alone for the treatment of otosyphilis. We conducted a retrospective study and included all consecutive patients diagnosed with otosyphilis who received only PGS treatment. The study period was from 2009 to 2013. The PGS treatment regimen was PGS 4 mu intravenously every four hours (24 mu/day) for 14 days. Clinical and audiogram outcomes were evaluated one year after treatment. There were 34 otosyphilis patients that were treated with PGS. After one year of treatment, 18 patients (52.9%) had a clinical improvement and 11 patients (32.4%) had an audiogram improvement. In conclusion, PGS at 24 mu/day for two weeks provided an audiogram improvement one year after treatment in one-third of the patients.

## 1. Introduction

Otosyphilis, caused by a spirochete *Treponema pallidum,* is a complication of syphilis and can occur in either early or late syphilis. The main symptom of otosyphilis is sensorineural hearing loss (SNHL) which can be found in 90.6% of otosyphilis patients. Other symptoms include tinnitus (72.9%) or vertigo (52.9%) [1]. The definite diagnosis of otosyphilis can be made by a positive inner ear fluid treponema test or a histological examination of the temporal bone. Neither method is practical in clinical practice. The diagnosis of otosyphilis therefore is usually made clinically by evidence of cochleovestibular dysfunction by audiogram and a positive result of treponemal tests. Other causes of cochleovestibular dysfunction must be excluded [2].

Early treatment of otosyphilis may improve hearing ability, while delayed treatment may result in permanent hearing loss. Previous studies used intramuscular benzathine penicillin G 2.4 mu/week for 3 weeks in patients with otosyphilis. The results were not promising [1,3]. A hearing improvement occurred in only 12.5–15% of patients [1,3]. Only 8 ears out of 58 ears (29 patients) or 15% had improved hearing function after one year of treatment [3]. The Centers for Disease Control and Prevention (CDC) recommends treating neurosyphilis with penicillin G sodium (PGS) 18–24 mu per day for 10–14 days [4]. There are, however, limited clinical data using PGS alone in otosyphilis patients.

## 2. Materials and Methods

This is a retrospective study at University Hospital, Khon Kaen University, Thailand. The study period was from 2009 to 2013. All consecutive patients diagnosed with otosyphilis were studied. The inclusion criteria were adult patients (age of over 15 years), having SNHL confirmed by an audiogram, a positive *Treponema pallidum* hemagglutination (TPHA) test in serum, and received only PGS 4 mu intravenously every 4 hours (24 mu/day) for 14 days. Patients were excluded if they had been diagnosed with Meniere’s disease, a tumor, trauma, chronic otitis media, toxic substances related to SNHL, HIV, or had received any previous treatment, such as doxycycline. 

Clinical data were retrieved from medical records, including baseline characteristics, duration of SNHL, cerebrospinal fluid (CSF) profile, CSF venereal disease research laboratory (VDRL) test, and treatment outcomes. The outcomes were defined clinically and by the audiogram test one year after treatment as improved, stable, or worse. The clinical evaluation was subjectively reported by the patients, while the audiogram improvement was defined by the pure tone average threshold at 0.5, 1, or 2 kHz if it decreased by more than 10 dB, if the pure tone average threshold decreased by more than 15 dB in every frequency, or the discrimination score increased by more than 15% [5]. 

The data are presented as the median (range) or number (percentage) for numerical and categorical data. Descriptive statistics were used to study the baseline characteristics and detect significant differences between the outcomes of the treatment. The factors associated with an audiogram improvement after one year were studied. A *p* value less than 0.05 was considered statistically significant. All the data analyses were performed by STATA software version 10 (StataCorp, College Station, TX, USA) on a personal computer. 

## 3. Results

During the study period, there were 34 patients who met the study criteria. Of those, 26 patients were male (76.5%). The median age of all patients was 71 years. Most patients had bilateral SNHL (24 patients; 70.6%), and the median duration of hearing loss was one year (Table 1). A hearing loss duration of more than one year was found in 20 patients (58.8%). The CSF analysis was performed in 30 patients (88.2%). Normal CSF profiles were found in 21 patients (70.0%) and the CSF VDRL test was negative in all patients.

After one year of treatment, 18 patients (52.9%) had a clinical improvement and 11 patients (32.4%) had an audiogram improvement, as shown in Table 2. There were no significant differences between the numbers of patients with clinical and audiogram outcomes (*p* value = 0.187). There were 14 patients with discordance between their clinical and audiogram outcomes (Table 3). The most common type of discordance was a clinical improvement and a stable audiogram outcome (7 patients; 20.6%). Clinical factors between those with and without an audiogram improvement after one year were comparable (*p* value > 0.05), including age, sex, sides of hearing loss, duration of hearing loss, and CSF profiles (Table 4). 

## 4. Discussion

There are several regimens in terms of the treatment of otosyphilis [1,3,5,6]. The two main treatment regimens are benzathine penicillin-based and PGS-based. Additional medications are probenecid and corticosteroid. Early studies found that benzathine penicillin was not effective in terms of hearing improvement [1,3]. Only 12.5% of patients had better hearing after one year of treatment with benzathine penicillin. Gleich et al. reported that an audiogram improvement was found in 5 out of 16 patients (31%) after treatment with the PGS-based regimen. PGS was given 24 mu intravenously for 10–14 days, depending on the CSF findings. Benzathine penicillin and probenecid and/or corticosteroid were added after the course of PGS, also according to the CSF findings [6]. The total course of treatment was long and may have been up to 118 days if the CSF profiles were abnormal. The overall audiogram improvement was 31%.

This study showed that the audiogram and clinical improvement one year after treatment was 32.4% and 52.9% after the two-week course of PGS treatment. The duration of treatment was shorter than the previous report but had comparable treatment outcomes [6]. No additional medications were added in this study. These findings suggest that additional medications may not be needed in the treatment of otosyphilis when treating with only PGS for two weeks. 

The efficacy of any regimen may not be favorable when patients have had a long duration of hearing problems (more than one year) prior to diagnosis and treatment of otosyphilis [6]. Out of 18 patients, 16 patients (88.9%) had hearing loss for at least one year and the longest duration was 20 years in two patients [6]. In this study, 20 patients (58.8%) had durations of hearing loss of more than one year resulting in an audiogram improvement in 32.4% of all patients, as shown in Table 2.

There are some limitations in this study. The small sample size and the retrospective design are the main limitations. Further studies to compare the PGS treatment with other regimens in a randomized trial are needed. Another limitation is that the otosyphilis in this study was not confirmed by the inner ear fluid treponema test or a histological examination of the temporal bone. All patients in this study were diagnosed with otosyphilis clinically. The advantage of this study is that it adds more data on the treatment outcomes of otosyphilis in non-HIV and Asian populations. Additionally, an objective evaluation of the hearing outcome was performed by using an audiogram.

## 5. Conclusions

In conclusion, the PGS 24 mu/day for two weeks provided an audiogram improvement one year after treatment in one-third of the patients.

## Figures and Tables

**Table 1:** Clinical features of the 34 patients with otosyphilis that were treated with penicillin G sodium.

Factors	Values
Age, years	71 (67–76)
Male, *n*	26 (76.5)
Sides of hearing loss	
Unilateral, *n*	10 (29.4)
Bilateral, *n*	24 (70.6)
Duration of hearing loss, year	1 (0.5–2)
Serum VDRL titer	
Non-reactive	4 (11.8)
Weakly reactive	9 (26.5)
1:1	7 (20.6)
1:2	8 (23.5)
1:4	4 (11.8)
1:8	2 (5.9)
Cerebrospinal fluid (CSF) profile	
White blood cell (cells/mm^3^)	0 (0–2)
Protein (mg/dL)	40.5 (33–56.5)

Please note: The data are presented as either the median (1st–3rd Interquartile rang, IQR) or the number of patients (percentage), and the CSF analysis was performed in 30 patients. VDRL: Venereal Disease Research Laboratory Test.

**Table 2:** Clinical and audiogram outcomes one year after treatment with penicillin G sodium treatment in otosyphilis patients.

Outcomes	Clinical *n* = 34	Audiogram *n* = 34
Improved, n	18 (52.9)	11 (32.4)
Stable, n	12 (35.3)	15 (44.1)
Worse, n	4 (11.8)	8 (23.5)

**Table 3:** Correlation between clinical and audiogram outcomes one year after treatment with penicillin G sodium treatment in otosyphilis patients (*n* = 34).

Clinical Outcome	Audiogram Outcome	Numbers (%)
Improved	Improved	10 (29.4)
Stable	Stable	7 (20.6)
Worse	Worse	3 (8.8)
Improved	Stable	7 (20.6)
Improved	Worse	1 (2.9)
Stable	Improved	1 (2.9)
Stable	Worse	4 (11.8)
Worse	Stable	1 (2.9)

**Table 4:** Clinical factors of patients with otosyphilis who were treated with penicillin G sodium, categorized by audiogram improvement one year after treatment.

Factors	Audiogram Improvement *n* = 11	Audiogram No Improvement *n* = 23	*p* Value
Age, years	70 (63–74)	72 (67–77)	0.35
Male, *n*	9 (81.8)	17 (73.9)	0.99
Sides of hearing loss			0.99
Unilateral, *n*	7 (30.4)	3 (27.3)	
Bilateral, *n*	16 (69.6)	8 (72.7)	
Duration of hearing loss, years	0.5 (0.5–4)	1 (0.5–2)	0.51
Cerebrospinal fluid profile			
White blood cell (cells/mm^3^)	0 (0–0)	0 (0–2)	0.35
Protein (mg/dL)	41 (33–46)	40 (34–58)	0.57
